# Acceptance, Sensory Characterization and Consumption Contexts for Dehydrated Persimmon Slices, Chips, Leathers and Powder: A Consumer Study

**DOI:** 10.3390/foods12101966

**Published:** 2023-05-12

**Authors:** Marina Castillo, Ana Pons-Gómez, Carlos Albert-Sidro, Barbara Delpozo, Cristina Besada

**Affiliations:** Sensory and Consumer Science Research Group, Postharvest Department, Valencian Institute for Agricultural Research, Crta Moncada-Náquera km 4.5. Moncada, 46113 Valencia, Spain

**Keywords:** discards, CATA questions, item-by-use, texture, taste, flavor, drivers of liking

## Abstract

Valorization of persimmon discards is a current challenge for the food industry. Obtaining dehydrated persimmon products can be a good option, but studies are necessary to predict consumer responses before placing new products on the market. In this study, we produced dried slices, chips, leathers and powder from persimmons that were discarded at harvest. A consumer study was performed with 100 participants. For a realistic context, the four products were presented to the participants in specifically designed packages to simulate commercial packages. The participants were asked about their interest in having each product available on the market. Then, they were asked to taste the samples and to state their acceptance and purchase intention. The participants characterized the main sensory properties of the samples using the CATA questions. The consumption contexts evoked by each product were also investigated based on the item-by-use method, plus the CATA questions. Our results revealed that, before tasting the samples, the participants showed special interest in having chips and slices available on the market. After tasting, the participants reported very good acceptance of chips, slices and powder, but leathers were less liked. According to the consumer characterizations, slices had the most intense persimmon taste and a succulent texture, while powder was characterized by its caramel taste. Chips were differentiated from the other samples, mainly for their crispy texture, while leathers were sticky and tasteless, which explained their poor acceptance. By evaluating the data on acceptance and the evoked consumption contexts together, we conclude that persimmon consumption could be enhanced by commercializing slices, chips and powder. The participants described chips and slices as healthy snacks in different daily situations, while powder could be used as a sweetener for yoghurts or hot drinks and as an ingredient for baking desserts. These are all contexts in which fresh persimmon would be not consumed as reported by the participants.

## 1. Introduction

Persimmon production has gained considerable importance in recent years in Spain, where the cultivation area has doubled in the last 10 years to cover 16,000 hectares. Spain is now the second most important producing country in the world after China [1,2]. This rapid increase in persimmon production volume has gone hand in hand with a notable increase in the volume of persimmon discards because all fruits that do not meet commercial standards are eliminated from the commercial chain. The percentage of discards during the harvest process and the postharvest period is estimated to be around 29% of total production [3]. Given this figure, one of the industry’s current challenges is to valorize discards.

In a recent study, Llorca et al. [4] obtained dried slices from persimmons that had been discarded for different reasons. The authors found that consumers liked the slices obtained from fruits discarded due to skin damages, malformations or chilling injuries as much as those obtained from the sound fruits that acted as the control. Previous studies have investigated how to obtain dehydrated persimmon products, such as dried persimmon slices [5,6,7,8], chips [6], powder [9,10] and leathers [11,12]. Some of these studies have addressed the optimization of product-obtaining conditions, but none has used discards as the raw material.

With this background, the above-mentioned study by Llorca et al. [4] opens the door for using persimmon discards as a raw material to obtain not only dried slices but also chips, powder and leathers, among other products. The ultimate goal is to provide the industry with options to valorize discards and to supply consumers with new persimmon products with a long shelf-life, which would allow them to enjoy persimmon when it is out of season. The first step to make this happen is to evaluate consumer interest in and acceptance of different products because the potential success of any products on the market will depend on these two factors to a great extent.

Valorizing persimmon discards would improve the industry’s sustainability and profitability. Furthermore, it could provide society with other benefits. Tarancón et al. [13] recently reported that the daily life contexts in which people consume fruits would be widened by the presence of fresh-cut and dehydrated fruits on the market. We herein wished to go one step further and evaluated if this would also be the case for a specific fruit with different dehydrated products. Thus, we hypothesized that the availability of different new dehydrated persimmon products (slices, snacks, leathers and powder) could enhance persimmon consumption by not only prolonging its commercial period but also increasing its daily consumption contexts.

Our hypothesis is also supported by different studies, which indicate that the ultimate decision to purchase or consume a product depends on both its intrinsic properties and the context of intended use (perceived situational appropriateness) [14].

The aim of this study was to determine consumer interest in and acceptance of different dehydrated persimmon products (slices, powder, leathers and chips) obtained from fruits discarded at harvest. The sensory properties of different products were described by the participants, and the drivers of liking were identified. We also investigated the evoked consumption contexts for each product and determined to what extent their availability on the market could contribute to the widening of persimmon consumption contexts.

## 2. Materials and Methods

### 2.1. Materials

‘Rojo Brillante’ persimmons (*Diospyros kaki* L.f.) discarded when they arrived at a packing house due to esthetic defects (skin damage, malformation and slight bruising caused by excessive pressure when picking) were obtained from a packing house located in Alcudia (Valencia, Spain). The fruits were transferred to the Valencian Institute for Agricultural Research (IVIA) (Moncada, Valencia) where they were submitted to standard CO_2_ deastringency treatment to remove astringency. After this treatment, the main physico-chemical parameters were determined in 10 fruits according to Novillo et al. [15]: color index (1000a/Lb de Hunter) of 19.2, firmness of 37.8 N, 17.5 °Brix and 0.04% of soluble tannins (f.w.).

Four different dehydrated persimmon-derived products were obtained: dehydrated slices, chips, leathers and powder. The method used to obtain each product is described below. Each method was based mainly on existing literature. The final conditions were set after performing some preliminary trials. The data on the water content of the final product are also cited below.

#### 2.1.1. Slices

Dehydrated persimmon slices were obtained following the conditions described by González et al. [8] with some modifications. The fruits were washed, peeled and cut transversely into 5 mm thick slices using an automatic luncheon meat slicer. The stalk and the opposite end were discarded. Hot-air drying was conducted in a cabinet dryer (Model FED 260 Avantgarde.Line, Binder GmbH, Tuttlingen, Germany) at an air velocity of 2 m·s^−1^ and a temperature of 60 °C for 11 h until the water content was 19.1 ± 1.1%.

#### 2.1.2. Chips

The chip process was the same as for slices, but the fruits were cut transversely into 1 mm thick slices using an automatic luncheon meat slicer without removing the peel, and a shorter dehydration time of 5 h at 60 °C was used. After this period, the water content was 12.04 ± 0.01%.

#### 2.1.3. Leathers

To obtain leathers, the method described by Dursun and Dalgıç [11] was followed with some modifications. The fruits were washed, peeled, cut into small pieces and blended in a blender for 2 min to obtain a smooth purée. This purée was mixed with water at a ratio of 75% purée to 25% water and was blended again for 1 min. The purée was spread on a metal tray to a thickness of 2 mm. The tray was placed inside the cabinet dryer for 5 h at 60 °C. The water content of this product was 17.14 ± 0.7%.

#### 2.1.4. Powder

Persimmon powder was obtained following the method of Yesilkanat and Savlak [16] with some modifications. The persimmons were cut into small pieces and placed inside the cabinet dryer at 80 °C for 32 h. After this time, the pieces were crushed by a grinder to obtain powder. Its water content was 9.4 ± 0.1%.

The obtained slices, leathers and powder were vacuum packed to preserve them until the sensory study was conducted. The chips were stored in a zip-lock bag. All products were left in a desiccator.

### 2.2. Consumer Study

One hundred participants formed part of the consumer panel and evaluated the persimmon-derived products. They were recruited in Valencia (Spain) through student mailing lists and by intercepting pedestrians on the street to invite them to participate in the study. Administration staff of the IVIA were also invited to participate as they were not linked to the food research field and could be considered naïve consumers. The participants’ age distribution was as follows: 41 participants were 18–30 years old, 38 were 31–50 years old, and 21 were more than 50 years old. Of the participants, 55 were women, 44 were men and 1 self-identified as non-binary. As a prerequisite for participation, it was necessary to be a regular persimmon consumer (at least once a month during the season).

**Figure 1:**
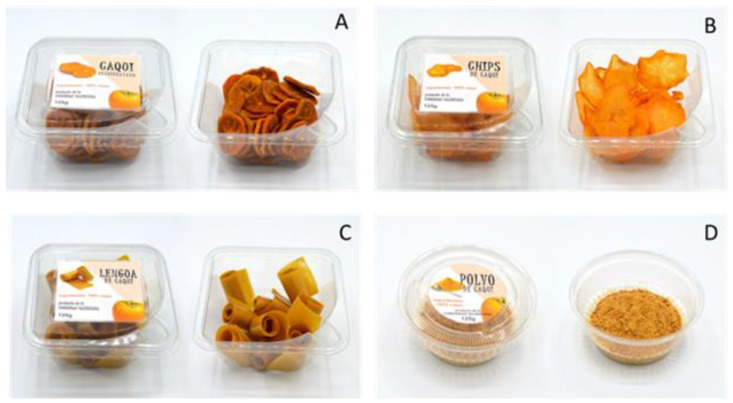
Products presented to the participants: (**A**) slices; (**B**) chips; (**C**) leathers; and (**D**) powder.

For a more realistic context, labels were specifically designed for each product in such a way that the samples were presented to the participants in a ‘commercial package’ (Figure 1). The four packages were monadically presented following a balanced Williams’ Latin square design.

The session was organized as follows:

(1) The packages with each product were presented to the participants one by one, who were asked to answer the following question before opening a package: ‘How much do you like the idea of being able to buy this type of product in supermarkets?’ Their answer was recorded on a 9-point scale, where 1 = ‘I think it is a bad idea’, 5 = ‘I am indifferent’ and 9 = ‘I like the idea very much’.

(2) Then, the participants were instructed to open the package and indicate how much they liked the sample’s appearance. After tasting the product, they had to indicate how much they liked its taste and texture, as well as their overall liking and purchase intention. A 9-point hedonic scale (1 = ‘I dislike it very much’ to 9 = ‘I like it very much’) was used to indicate their acceptance, and a 5-point scale (1 = ‘I would certainly not buy it’ to 5 = ‘I would certainly buy it’) was employed to indicate their purchase intention. Next, they were asked to answer the Check-All-That-Apply question (CATA question), which comprised 22 descriptors related to taste and texture characteristics. To this end, they were given the following instructions: ‘Taste the sample and check all the attributes from the list that apply to it’. The repertory grid method was used to generate the list of attributes. The samples were presented in triads to the members of a semi-trained panel consisted of 8 panellists, who were asked to describe all the similarities and differences that they perceived within each triad. Those descriptors referred to most frequently and with the greatest consensus among the panel members were included in the attribute list: ‘not much taste’, ‘intense taste’, ‘not very sweet’, ‘sweet’, ‘very sweet’, ‘unrecognizable persimmon taste’, ‘slight persimmon taste’, ‘intense persimmon taste’, ‘caramel taste’, ‘persistent taste after swallowing sample’, ‘soft’, ‘firm’, ‘succulent’, ‘easy to chew’, ‘difficult to chew’, ‘crispy’, ‘melts in the mouth’, ’floury’, ‘grainy’, ‘elastic’ and ‘sticky’.

(3) As a final task, the participants responded to an item-by-use test using a CATA question. This task was designed to evaluate the main consumption contexts evoked by the participants for each product. During this task, the participants were also asked about fresh persimmon consumption contexts. The question was formulated as follows: ‘In which situations would you eat, or would you think it is appropriate to eat, this sample?’ The list included 17 consumption contexts: ‘As part of breakfast or lunch’, ‘As a dessert with lunch or dinner’, ‘As a children’s lunch at school’, ‘As a healthy alternative to traditional snacks’, ‘As an ingredient for baking desserts or pastries’, ‘As an ingredient in dishes (salad, sauce, etc.)’, ‘To sweeten hot drinks (coffee, milk, tea, etc.)’, ‘To sweeten yoghurt’, ‘To prepare juice/smoothies’, ‘As a snack at home’, ‘As a snack to eat when I’m not at home (at work, university, traveling)’, ‘When I feel like eating a sweet snack, but want it to be healthy’, ‘As something to eat when I have a drink (soft drink, beer, etc.)’, ‘As a convenient and quick way to eat fruit’, ‘As a healthy alternative to sweets for children’, ‘To take on a picnic or trip’ and ‘When I’m practicing sport’.

(4) In the final section, the participants answered demographic questions about gender and age.

The questionnaire was designed using the Fizz software (Fizz Acquisition 2.51). The participants responded using tablet devices.

The protocol and procedures for this study were reviewed by the Scientific Directorate of the IVIA, which requested a waiver consent. All the guidelines of the Declaration of Helsinki and the 2016/679 EU Regulation on the protection of natural persons with regard to the processing of personal data and the free movement of such data were met. Consent indicating voluntary participation was obtained from all participants. To this end, at the beginning of the questionnaire, they were shown the following statement: ‘If you agree to participate and us to employ your answers for this study, please click next’. When the participants finished the questionnaire, they received a gift for taking part in the study.

### 2.3. Statistical Analysis

The Kruskal–Wallis test, followed by Dunn’s multiple comparison test, was applied to evaluate differences in the acceptance scores among the samples (*p* ≤ 0.05).

The frequencies of mentioning each attribute in the CATA question were determined for each sample. The non-parametric Cochran’s Q test was performed on the raw binary CATA data to determine significant differences among the samples for each sensory attribute (*p* ≤ 0.05). Then, a correspondence analysis (CA) was carried out on the frequencies of mentioning the contexts for each sample to visualize the most relevant differences among the samples according to the sensory properties/consumption appropriateness as perceived by the participants.

A penalty analysis of the CATA data was used to determine the attributes with an impact on acceptability. For each attribute, the difference in the average liking of those products with the attribute and those without it was calculated.

All analyses were carried out using the XLSTAT software (version 2023, Addinsoft Inc., New York, NY, USA).

## 3. Results and Discussion

### 3.1. Consumer Interest in Each Persimmon-Derived Product, Acceptance and Sensory Characterization

To successfully launch new food products that appeal to consumers in both health and hedonistic terms, it is important to determine how people perceive such foods to better understand consumer choice behavior. Thus, industry and research communities are increasingly advocating for a consumer-oriented approach to new product development. The development of a new food concept is one of the earliest stages of this process during which consumer participation proves extremely valuable. Later, another crucial product development step is prototype tasting, which may help to optimize its formulation [17].

As explained in the Introduction section, different research studies have addressed how to obtain the four persimmon-derived products herein evaluated. Consumer prototype tasting was performed in some of these studies on slices or leathers [6,8,11]. With regard to powder, an existing consumer study evaluated it as an ingredient to replace sugar in gluten-free cakes in such a way that consumers tasted the cakes and not the powder itself [16]. To date, no studies have jointly evaluated consumers’ responses to slices, chips, leathers and powder. Furthermore, no study has asked consumers to state how appealing they find the idea of having these products on the market. Thus, in the present study, before allowing the participants to taste the samples, they were asked how interesting they found the idea of being able to buy each product.

The participants’ responses revealed that they very much liked the idea of having chips and dried slices available and gave scores of around 8 on a 9-point scale. The participants showed less interest in leathers and powder and scored the idea of their availability at around 6.7.

As explained in the previous section, after the participants tasted the samples, they were first asked about their acceptance and purchase intention. Then, they performed the sensory characterization using the CATA question task. This is the recommended task order for sensory studies [18], which we followed. However, to more fluently discuss the results, we first presented the sample characterization results and then the results for acceptance.

**Figure 2:**
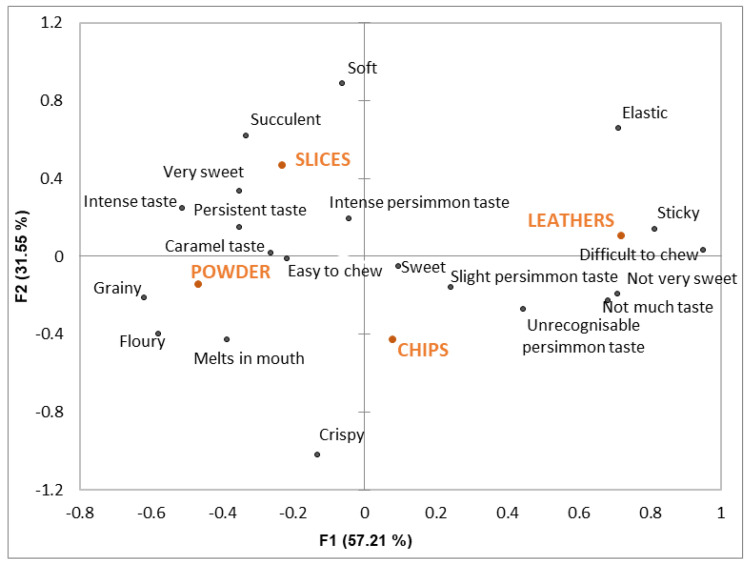
The correspondence analysis performed with regard to the CATA attributes. Samples are denoted by capital letters and attributes are depicted by lowercase letters.

Figure 2 shows the correspondence analysis between the samples and descriptors. The first and second dimensions explain almost 90% of the variability. This indicates that the attributes on the CATA list allowed the participants to describe the sensory differences that they perceived among the samples. ‘Firm’ is the only attribute that does not appear in the figure because its contribution is not statistically significant.

Leathers are allocated to the right of the figure and separately from the other samples. They are associated mainly with texture attributes, such as sticky, elastic and difficult to chew. For this sample, the participants indicated a lack of taste and not very sweet. The powder and slice samples are allocated to the opposite part of the figure and are described as having a very sweet, intense and persistent taste. The flavor and texture attributes help to differentiate these two samples. Powder is described as having a caramel flavor, melting in the mouth, and having a grainy and floury texture. Slices are the samples with the most intense persimmon taste and have the softest and most succulent texture. Chips are allocated to the bottom of the figure, described as having a sweet taste and are characterized mainly by their crispy texture.

All persimmon products were submitted to a dehydration process, but the geometry of the samples and the final water content differed between them, which would explain the differences in texture and taste that the participants described.

The samples with the highest water content were slices, and their texture was defined as the softest and most succulent. The very different texture of leathers, described as being sticky and difficult to chew, could be related to the fact that they were obtained from purée and, therefore, the original parenchyma structure was completely lost.

The chips were thinner than the slices and had a lower water content, which justifies their crispy texture. In fact, this was the most relevant attribute for the chips’ characterization. Crispy is defined by Fillion and Kilcast [19] as “a light and thin texture producing a sharp clean break with a high-pitch sound when a force is applied, mainly during the first bite with the front teeth”. This definition clearly explains why powder was not characterized as crispy despite it having the lowest water content of all the samples. The main attributes used by the participants to define the powder’s texture were actually related to its particulate geometry (grainy and floury) and melting properties.

Regarding taste and flavor, dehydration implies changes that are linked mainly with the concentration of sugars and with the changes and loss in aromas, along with the possibility of new aromas being developed [20]. Slices were the samples with the most intense persimmon flavor, which is likely related to their less intense dehydration as revealed by their final water content. It is also worth mentioning the caramel flavor that developed in the powder samples. Farina et al. [21] reported that a trained panel found a honey and caramel odor in hot air-dried loquats. These authors explained that this fact was due to partial sugar caramelization. Along the same lines, after adding persimmon powder to gluten-free muffins, Hosseininejad et al. [10] reported an increase in sweetness, a fruity and caramel taste, and a dark color as the main sensory changes compared to the control sample with no added powder.

Finally, the leather samples’ lack of flavor could be because we had to add a certain proportion of water during processing to make the purée more fluid and to spread it on the trays. This might explain the lack of flavor due to dilution and suggests the need to optimize the obtained conditions. To the best of our knowledge, only one study has reported leathers obtained from persimmon fruit [11]. That study did not specify either persimmon variety or fruit maturity, which are two factors that can substantially affect both the purée and the obtained leather properties. Based on our results, it is likely that using soft persimmon as the raw material would provide a sweeter and more fluid purée, and perhaps the step of having to add water could be avoided.

**Figure 3:**
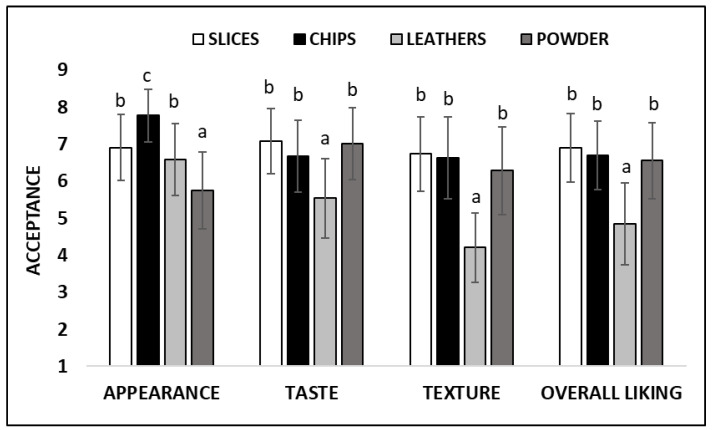
Consumer acceptance of the different attributes for the four dehydrated persimmon products. The mean values ± standard deviation are shown. Scale: 1—’I dislike it very much’ to 9—’I like it very much’. For each attribute, the scores that do not share the same letters are significantly different (*p* ≤ 0.05) according to the Kruskal–Wallis test.

Figure 3 shows the results of consumer acceptance of the different sample attributes (appearance, flavor and texture), and their global acceptance, taking into account all the consumer perceptions of the products.

Our results showed that the participants liked the appearance of the chips the best, followed by that of the slices and leathers. Persimmon powder was visually less appealing.

When tasting the samples, the participants really liked the flavor of slices and powder, i.e., their intense and persistent taste with the persimmon and caramel flavor, respectively. No differences in liking scores were detected for chips, which sweet and light persimmon flavor was widely accepted by the participants. Leathers obtained a different response because the participants liked them significantly less (liking scores < 6).

Despite slices and chips having very different texture properties (Figure 2), when the participants were asked about texture acceptance, they gave high and equal scores to both samples. Powder texture was also well accepted by the participants. In terms of flavor, leathers received the lowest texture scores for being sticky and difficult to chew.

As a final step for the tasting task, the participants were asked for their global evaluation of the samples by considering all of their perceptions (global liking). The participants’ favorite products were slices and chips, followed closely by powder. In accordance with their lower flavor and texture acceptances, leathers also received a significantly lower overall rating.

**Figure 4:**
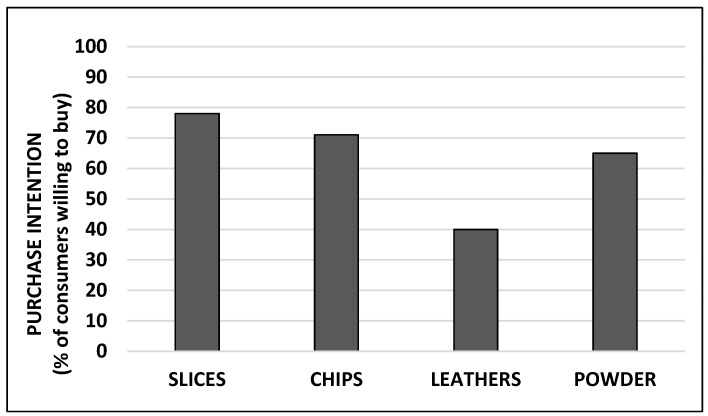
Purchase intention for different persimmon products expressed as the percentage of participants who stated that they would certainly/probably/perhaps buy them.

This pattern of consumer product preferences was reflected in their purchase intention (Figure 4). Slices and chips were the samples with the highest percentage of purchase intention, with 70–80% of the participants willing to buy them. The purchase intention for the powder samples was slightly lower (65%), and only 40% of the participants stated that they would buy leathers.

**Figure 5:**
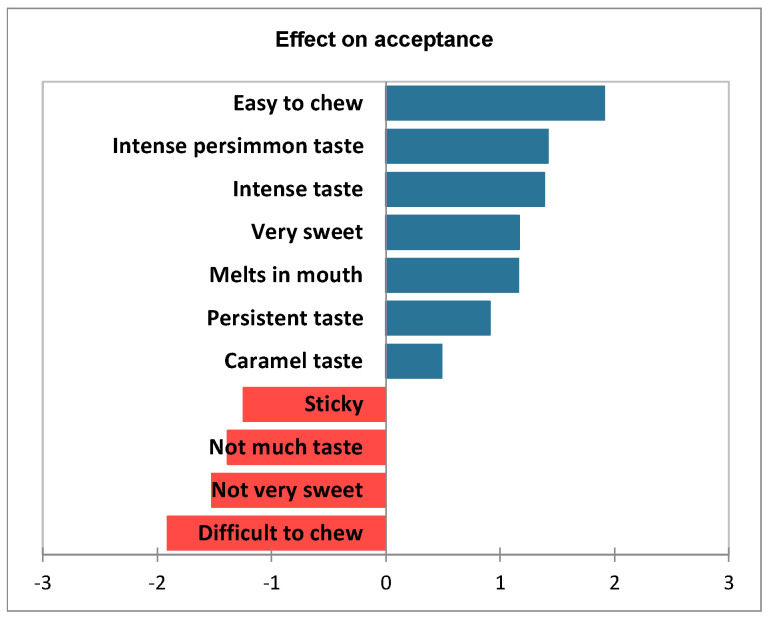
Penalty analysis: impact on the average liking scores. Only attributes with a significant impact on liking (*p* ≤ 0.05) are included. Blue bars indicate the increment of the mean acceptance score linked to those attributes with a positive impact. Red bars indicate the reduction in mean acceptance score linked to those attributes with a negative impact.

To acquire more information from the data, a penalty analysis was performed to clearly visualize which attributes positively contributed to acceptance and which had a negative impact. As observed in Figure 5, the drivers of liking for the dehydrated persimmon samples are linked to chewiness and flavor intensity. On the one hand, ‘easiness to chew’, ‘intense persimmon taste’, ‘intense taste’ and ‘very sweet’ are the attributes that contribute the most to liking. On the other hand, ‘difficult to chew’, ‘not very sweet’, ‘not much taste’ and ‘sticky’ are the attributes that contribute negatively to liking.

### 3.2. Consumption Contexts for Fresh Fruit and Products

One of the objectives of this study was to investigate to what extent the persimmon-derived products would contribute to widening the contexts in which people would consume persimmons. The participants were asked about the contexts for consuming the four evaluated samples and for fresh persimmon. As being a regular persimmon consumer was a prerequisite to participate in this study, we did not need to provide participants with a fresh fruit sample.

**Figure 6:**
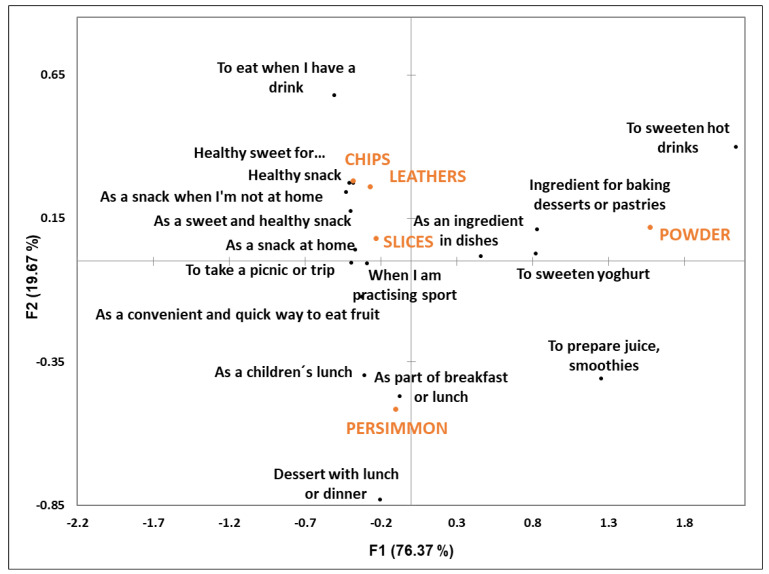
The correspondence analysis performed with regard to the CATA contexts. Samples are denoted by capital letters and contexts are depicted by lowercase letters.

Figure 6 shows the results from the CA performed between the samples and the consumption contexts. Fresh persimmon is allocated at the bottom left of the figure and is associated mainly with eating it as a dessert after meals or at breakfast, and as a children’s snack. This result falls in line with Tarancón et al. [13], who investigated the consumption contexts evoked by different fresh fruit types (easy-to-peel, big-sized fruits; small-sized fruits; and fruits that need cutlery). They found that big-sized fruits and fruits that need cutlery, such as persimmon, are more suitable to be eaten as part of the main meals at home.

Slices, chips and leathers are allocated to the upper part of the CA figure and are very close to one another. This means that these three samples share the same consumption contexts as evoked by the participants. These contexts are linked with the samples’ healthy and sweet properties; these three samples are considered appropriate to be eaten as snacks (‘a healthy sweet for children’, ‘a healthy snack’, ‘as a snack when I’m at home’, ‘to eat something sweet and healthy’, ‘as a snack when I’m not at home’, ‘to take on a picnic or trip’, and ‘as a convenient and quick way to eat fruit’). Chips are highlighted as something to eat when having a drink, while slices are considered appropriate to take on a picnic, to eat when practicing sports, and as a convenient and quick way to eat fruit. These specific contexts for chips and slices are probably linked with their texture and convenience. In Spain, when going out to have a drink at a bar, it is normal to be offered potato chips or peanuts to eat with the drink. It is, therefore, likely that the crispy texture of these products has led the participants to associate crispy chips with this specific context.

Additionally, it is known that the dehydration process generally increases fruit consumption convenience [22]. This is evidenced by persimmons and other fruits that, when eaten fresh, need to be prepared beforehand (peeling and cutting with a knife). Different authors have reported that food consumption convenience is more relevant for snacks and away-from-home contexts than main meals at home [13,23]. Accordingly, our results showed that the participants chose fresh fruit to be eaten at home, while most of the participants chose the dehydrated samples (chips, slices and leathers) to eat when not at home.

Finally, powder appears far from the other products on the right side of the CA figure. As revealed by the participants’ characterization of the products, the powder characteristics are completely different from those of the other samples, as are its consumption contexts. Powder was perceived as an ingredient for desserts or pastries and as a sweetener for hot drinks or yoghurt. In a recent review about the influences of dried fruit and vegetable powder on cake quality, Perfilova et al. [24] reported that the powder of fruits and vegetables exhibited benefits in attracting consumers by improving the appearance, texture, nutritional values, sensorial properties and shelf life of cakes. Specifically for persimmon powder, Hosseininejad et al. [10] reported that its addition acted as a functional ingredient to gluten-free muffins. These authors used persimmon powder to partially replace corn flour and described an increase in carotenoids and antioxidant capacity. As previously mentioned, they reported that the added powder increased not only sweetness but also fruity and caramel tastes, and a darkened muffin color.

The CA between the samples and the evoked consumption contexts revealed that the contexts chosen mainly by the participants for eating fresh persimmon differed from those evoked for eating the persimmon-derived samples. This result suggests that daily persimmon consumption contexts could be widened by persimmon-derived products when available for consumers. To corroborate this result, we studied the data in more detail by taking into account the participant factor. For this purpose, we calculated the percentage of participants who would not eat fresh persimmon in each context but would consume any of its derived products. As the CA revealed that chips, leathers and slices shared most of the contexts, these three samples were merged to form a single group, which we called ‘snack products’. Thus, if a participant had checked more than one of these ‘snack products’ (chip, leathers and slices), only one count was considered. The results of this analysis are shown in Table 1. For each context, the first column indicates the percentage of participants who would extend their consumption by eating any of the snacks. The second column denotes those who would do so by selecting powder.

The results of this analysis are quite revealing because, in all the contexts, a certain percentage of participants would potentially eat the persimmon-derived products, but not fresh persimmon. There are eight contexts for the snack products that are highlighted because their percentage is higher than 30%: ‘healthy alternative to sweets for children’, ‘a healthy alternative to traditional snacks’ and ‘a snack when not at home’ are endorsed by close to 50% of participants, and ‘as sweet and healthy snack’, ‘to eat when having a drink’, ‘a snack at home’, ‘an ingredient for baking desserts’ and ‘for a picnic or trip’ have between 30% and 40% of participants. For powder, three contexts are worth noting: ‘sweetening hot drinks’, ‘ingredient for baking desserts’ and ‘sweetening yoghurt’.

Our results show that the several products produced in this work cover different consumption contexts. These results strongly suggest that it is possible to increase persimmon consumption by offering consumers dehydrated products with different sensory properties, which are also convenient and suitable to be eaten in distinct daily life scenarios.

According to Arvola et al. [25], one main barrier to eating healthy snack products is that consumers associate these food options with poor taste and a less pleasurable eating experience. In the same line, Crofton and Scannell [26] concluded that the next major step for healthy snack manufacturers is to strengthen the link between hedonic and healthy dimensions by utilizing flavor and texture to create tasty products that are healthy. Our results showed that the study participants very much liked the chips and slices, and they considered these two products to be a good snack option. The information presented in this work may be key for the industry to overcome this “poor taste” barrier. Thus, the range of health-, sensory- and convenience-related benefits associated with these two snacks should be taken into account when designing future communication strategies for these products. This information can be helpful when creating marketing strategies and might positively affect their adaptation and diffusion, which will eventually contribute to greater persimmon fruit consumption.

## 4. Conclusions

In this study, four different dehydrated persimmon-derived products were obtained: chips, slices, leathers and powder. The consumer characterization of these samples by means of CATA questions revealed that slices had the most intense persimmon taste and a succulent texture, while powder was characterized by its caramel taste. Chips were differentiated from the other samples, mainly for their crispy texture, while leathers were sticky and lacked persimmon taste, which explained their low consumer acceptance. According to our results, commercialization of slices, chips and powder would be very well accepted by consumers. Moreover, the availability of these products on the market would very likely extend persimmon consumption. The participants in this study, who were all adults, stated that persimmon slices and chips could be a healthy option when feeling like eating a sweet snack, and they thought that these two persimmon snacks would be a good alternative to less healthy sweets for children. Thus, future research should address and explore children’s responses to persimmon snacks as they may be potential consumers. Storability is also a key aspect to address before launching these persimmon products on the market. Preserving drivers of liking, such as the crispness of chips or the texture characteristics of powder, could be challenging.

## Figures and Tables

**Table 1:** Percentage of consumers who believe it is suitable to consume the dehydrated products and powder in the proposed contexts but do not consider it appropriate to consume fresh fruit.

	NO Fresh– YES Snacks (%)	NO Fresh– YES Powder (%)
Breakfast or lunch	8	4
Dessert at lunch or dinner	2	0
School lunchbox	20	0
Healthy alternative snack	**49**	3
Ingredient for cooking desserts	**32**	**50**
Ingredient in dishes	26	25
Sweetening hot drinks	19	**61**
Sweetening yoghurt	20	**44**
Prepare juices or smoothies	4	28
Snack at home	**33**	2
Snack when not at home	**49**	0
Sweet and healthy snack	**47**	1
To eat when having a drink	**37**	3
Convenient and quick way to eat fruit	30	1
Healthy alternative to sweets for children	**54**	0
Picnic or trip	**31**	0
Practice sport	24	0
Breakfast or lunch	8	4

## Data Availability

Data presented in this study are available from the corresponding author upon request.
